# Using Spectral Flow Cytometry for CAR T-Cell Clinical Trials: Game Changing Technologies Enabling Novel Therapies

**DOI:** 10.3390/ijms251910263

**Published:** 2024-09-24

**Authors:** Thomas C. Beadnell, Susmita Jasti, Ruqi Wang, Bruce H. Davis, Virginia Litwin

**Affiliations:** 1 Eurofins Viracor Biopharma, Lenexa, KS 66219, USA; thomas.beadnell@vbp.eurofinsus.com (T.C.B.); susmita.jasti@vbp.eurofinsus.com (S.J.); 2 Eurofins Pharma Bioanalytical Services, St. Charles, MO 63304, USA; ruqi.wang@bcl.eurofins.com; 3 Independent Researcher, Clifton, ME 04428, USA; davis.bruce@gmail.com; 4 Eurofins Clinical Trial Solutions, Montreal, QC J2L 3N5, Canada

**Keywords:** spectral flow cytometry, CAR T-cell, immunogenicity, clinical trial, CLSI H62, NIST Flow Cytometry Standards Consortium

## Abstract

Monitoring chimeric antigen redirected (CAR) T-cells post-infusion in clinical trials is a specialized application of flow cytometry. Unlike the CAR T-cell monitoring for individual patients conducted in clinical laboratories, the data generated during a clinical trial will be used not only to monitor the therapeutic response of a single patient, but determine the success of the therapy itself, or even of an entire class of therapeutic compounds. The data, typically acquired at multiple testing laboratories, will be compiled into a single database. The data may also be used for mathematical modeling of cellular kinetics or to identify predictive biomarkers. With the expanded context of use, a robust, standardized assay is mandatory in order to generate a valuable and reliable data set. Hence, the requirements for assay validation, traceable calibration, technology transfer, cross-instrument standardization and regulatory compliance are high.

## 1. Introduction

### 1.1. CAR T-Cells

Chimeric antigen redirected (CAR) T-cells are genetically re-engineered to enhance the killing of a specific target cell independent of the normal T cell receptor (TCR)/CD3 machinery. This is accomplished by T cell transduction with constructs that express the antigen binding fragments of antibody heavy and light chains connected by a linker creating a Single Chain Variable Fragment (ScFv). The ScFv is then connected to a transmembrane domain and intracellular co-stimulatory and signaling domains.

This novel category of living drugs is proving to be one of the most impactful innovations of modern medicine. Initially applied as immunotherapy for the treatment of CD19 positive, B-lineage leukemia and lymphoma, they are currently being evaluated for the treatment of non-hematologic malignancies and autoimmune conditions as well. Despite their remarkable initial successes, post-infusion events, such as changes in CAR T-cells from activated to exhausted phenotypes, as well as the impact of inhibitory responses from the endogenous immune system, result in altered efficacy [1,2].

The first-generation CAR T-cell targeted a single antigen. In order to overcome some of the observed resistance mechanisms, the next generation of constructs target multiple antigen targets. There are four categories of dual CAR constructs: two single transductions, dual transductions, bi-cistronic-CAR transductions and bi-tandem-CAR transductions [2]. Single transduction dual CAR techniques use two separate single targeted CAR T-cell products. These therapies are generated by transducing independent T cells with two independent single CAR T vectors. The products are then pooled together prior to infusion, or independently administered. Dual transduction CAR techniques also use two separate CAR T-cell products; however, they are used to transduce the same T cells and are administered as one therapy. Bi-cistronic-CAR T-cells are transduced with one vector containing dual expression cassettes, resulting in the expression of two independent single-CARs. Bi-tandem-CAR T-cells are transduced with one vector containing one expression cassette resulting in the expression of a bivalent dual targeting tandem CAR. Dual-targeting strategies are being evaluated in Relapsed/Refractory Multiple Myeloma (RRMM), which requires the blocking of multiple surface proteins to successfully induce a durable response [3].

### 1.2. Spectral Cytometry

Recently a 50-color immunophenotyping panel characterizing human T cell subsets and dendritic cells was published [4]. The ability to achieve such high parameter measurements is due, in large part, to the advancement of spectral flow cytometry as well as novel fluorescent probes [5].

Conventional flow cytometers capture only a portion of the fluorescent output from the probes using as series of long pass, short pass and band pass filters. Using a one fluorophore per detector model, conventional flow cytometers are limited to measuring only fluorophores, which can be detected by the instrument’s specific configuration of filters in combination only with those fluorophores with distinct, non-overlapping emission peaks and spillover into other channels that can be reasonably compensated. Thus, the number of fluorophores which could be combined in a single panel, and hence the number of cellular attributes evaluable, became a limiting factor.

In the early 2000s, J. Paul Robinson and colleagues highlighted the future of flow cytometry and the requirements for moving towards multispectral flow cytometry, driven by the need for collecting an increasing number of cellular variables [6]. The perspectives piece was subsequently followed up by a demonstration of the capability in 2012, and the ability to perform multispectral analysis through the collection of signals in 32 channel detectors [7]. This advancement was followed in 2015 by the first commercial spectral cytometer released by Sony [8].

Advances in the instrumentation, detectors and unmixing algorithms (supported by the advancements in computing power) allowed the field to take better advantage of the full spectrum of fluorophores. The use of prisms and grating of light followed by the spectral unmixing of the signal takes advantage of the full spectrum of the fluorophore, such that each fluorophore is no longer identified merely by the emission peak, but rather a unique fingerprint of the emission of the fluorophore across the measured spectrum. The result is more fluorescent probes can be evaluated in a single panel and probes with overlapping primary emissions peaks can be used in the same panel, given any uniqueness in their full spectrum signature.

A key difference between conventional and spectral instruments is the importance of an increase in detectors/channels on the spectral instruments to allow for better mapping of the spectrum of the fluorophore. Spectral analysis can also lead to better standardization, since it removes the variability and subjectivity inherent in establishing instrument compensation settings, particularly if the assays integrate bead or autologous cell calibrators.

### 1.3. The Intersection of Clinical Need and Technilogical Advances

In the 1980s, early in the acquired immune deficiency syndrome (AIDS) pandemic, the monitoring of CD4 T-cell counts became an essential part of AIDS diagnosis. When human immunodeficiency virus type 1 (HIV-1) was identified as the causative agent of AIDS, and the first clinical trials for HIV anti-viral therapies were conducted, CD4 T-cell counts were part of the enrollment criteria for the trials. After U.S. Food and Drug Administration (FDA) approval of azidothymidine (AZT), the first drug for treating AIDS, decisions regarding when to begin anti-retroviral therapy were based on CD4 T-cell counts [9].

Initially, assays for CD4 T-cell counts were measured using fluorescent microscopy and manual cell counting. Shortly thereafter, as flow cytometry matured as a technology, CD4 T-cell absolute counts were conducted by flow cytometry. Around the same time, highly specific flow cytometric methods, able to distinguish CD4+ peripheral blood monocytes from CD4+ T-cells, became available. Advances in the technology and the sophistication of the methodology for measuring CD4 T-cell absolute counts were in part driven by medical need. Assays transitioned from fluorescent microscopy and manual cell counts to highly specific flow cytometric methods that were able to distinguish CD4+ peripheral blood monocytes from CD4+ T-cells [10].

Around this time, the first standards for flow cytometric methods were developed by Janis Giorgi, Frank Mandy, Jan Nicholson and other members of what is now the International Society for the Advancement of Cytometry (ISAC). The Multicenter AIDS Cohort Study (MACS) initiated in 1984 was the first clinical interlaboratory study (ILS) for clinical flow cytometry. The study not only identified the best practices for attaining comparability of lymphocyte subset determinations in longitudinal, multicenter studies, but highlighted the value of establishing reference intervals in control populations when interpreting patient data sets. Ultimately, the study results led to the first quality control programs in flow cytometry laboratories [11].

Following the example set some 40 years ago by MACS, the National Institute of Standards and Technologies (NIST) has launched the Flow Cytometry Standards Consortium (FCSC) to help develop the measurement assurance solutions and standards needed for generating high quality flow cytometric results supporting cellular therapies [12]. The NIST FCSC serves as a neutral forum for stakeholders from industries, government agencies, academia and other organizations to identify and address common challenges, share best practices and accelerate the development of standards and reference materials towards quantitative flow cytometry [13]. The NIST FCSC has just completed two of the largest ILS to date, which included 50 instruments and 19 different institutions. The first ILS focused on instrument setup and standardization, while the second focused on the major immunophenotype of the major lymphocyte populations. The results from these ILS are expected to be published in the coming year. Soon, another ILS will be initiated for the evaluation of CD19-specific CAR T cells.

The dependency of a novel technology and the clinical evaluation of novel therapeutic modalities appear to be repeating themelves today with the advances in both cellular therapies and spectral cytometry. It is not surprising that flow cytometry, the premier technology for single-cell characterization, is critical to all phases of the design and development of CAR-T cells, a unique class of therapies where the drug compound is composed of a heterogeneous mixture of living cells.

The advantages of spectral flow cytometry over conventional cytometry are primarily due to the fact that higher-parameter assays are achievable. High parameter assays are essential when deep phenotyping is required while at the same time specimen volumes are limited, as is the case in clinical trials for cellular therapies.

At various time points in a clinical trial, the cells reported from the flow cytometric method will be considered as rare events. As described in more detail below, after administration of immunodepleting therapy, endogenous immune cells will be rare, the circulating CAR T-cells will also be rare at various timepoints post-infusion, and the leukemic cells will be rare when assessing measurable residual disease (MRD). The highly specific and sensitive assays required for rare event detection can be more easily delivered by higher parameter, spectral methods compared to conventional methods [14,15]. High parameter assays facilitate increased assay specificity by allowing for additional negative and positive selection antigens to be included in the same staining and acquisition [14,15]. With the ability to subtract auto-fluorescence, spectral cytometry panels have the potential be more sensitive than those developed for conventional cytometers given that spectral cytometry allows for better detection of dim antigens.

## 2. Critical Measurements during CAR T-Cell Clinical Trials

Guidance from EMBTA/EHA for the primary study objectives for CAR T-cell therapy targeting a B cell hematological malignancy typically include cellular kinetics (CK) to track the distribution, expansion, contraction and persistence of the CAR T-cells [16]. Additionally, clinical outcomes will include measurable residual disease (MRD) and the duration of disease-free survival (time to relapse).

While it is known that anti-tumor efficacy and long-term remission rely on prolonged CAR T-cell persistence and expansion, other parameters influencing sustained relapse-free survival vs. resistance to therapy have not been fully delineated [1,17,18]. In a clinical trial setting, it is not sufficiently informative to enumerate the circulating levels of CAR T-cell and malignant B cells; expression levels of both the CAR T antigen on the effector cells and the target antigens on leukemic cell populations should also be quantitatively measured, as they will influence CAR T-cell efficacy and persistence [19,20,21]. In addition, extensive immunophenotyping of the CAR T-cells should be conducted to enable assessment of CAR T-cell in vivo stability post-infusion. The specimen type is dependent on the disease state, COU and the study objectives. Typically, the matrix would be peripheral blood or bone marrow.

Deep immunophenotyping of the CAR T-cells along with the endogenous immune cells are helping to shape our understanding of the cellular characteristics that can predict prolonged remission. Using high-parameter, spectral flow cytometry, most, if not all, of the measurements for the CAR T-cells, leukemic cells and endogenous immune cells could conceivably be evaluated in a single high-parameter panel. This approach would generate high quality data and reduce the sample requirements, which is critical during clinical trials.

The procedures for developing complex, high-parameter, spectral flow immunophenotyping methods are now well established [22]. Processes for the validation and monitoring of flow cytometric methods are also now well established and described in the recent guidance document issued by the Clinical and Laboratory Standards Institute (CLSI), H62- Validation of Assays Performed by Flow Cytometry [23]. Testing conducted for use in clinical trials has a unique context-of-use (COU) compared to testing in clinical testing laboratories. Data may be used to make decisions regarding patient care and treatment, for a new drug application (NDA) or biologics license application (BLA) regulatory submission, or to define previously unknown correlates of clinical response. All assays should be validated appropriately for the intended use. CLSI H62 describes validation protocols appropriate for various COUs of flow cytometry. As elaborated in CLSI H62 and a publication by Sommer et al., the development and validation of flow cytometric methods detecting and reporting cell subsets that are found in low frequency, so called “rare events”, have unique requirements [14,23]. These assays need not only have highly sensitivity in order to reproducibly report low numbers of events in the final gate, but they also need to be highly specific in order to make sure the events in the gate are what are intended and not another cell type or debris [14,23]. The lower limit of detection (LLoD) and lower limit of quantification (LLoQ) must be validated as described in CLIS H62 and the testing procedures must describe how results below the LLoQ will be reported.

### 2.1. Extensive CAR T-Cells Immunophenotyping

**Figure 1:**
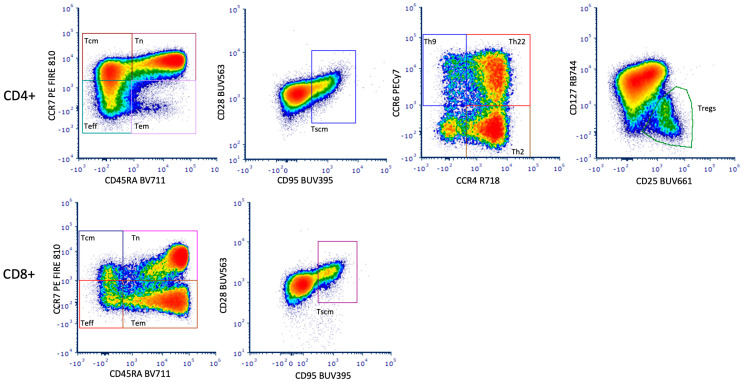
Representative staining from a 26-color immunophenotyping panel for T cell characterization. Cryopreserved human PBMC were stained with directly conjugated mAb and acquired on a Becton Dickinson FACSymphony A5 Spectrally Enabled flow cytometer. This instrument is capable of both conventional and spectral cytometry. The data represented here were acquired using the spectral mode. Data were unmixed post-acquisition. Naïve (Tn), Stem-like Memory T cells (Tscm), Central Memory T cells (Tcm), Effector Memory T cells (Tem), Effector T cells (Teff), T Regulatory Cells (Tregs).

An important objective for CAR T-cell clinical trials is to identify any correlates of CAR T-cell duration, target antigen density and anti-tumor activity. An understanding of the ability of the CAR T-cells to provide prolonged remissions is being shaped by the CAR T-cell phenotypic characteristics. Phenotyping panels should include markers for T cell maturation, activation, activation induced cell death, homing and exhaustion/senescence (see Table 1, Table 2 and Table 3 and Figure 1).

The anti-tumor activity of CAR T-cells relies on prolonged persistence and expansion and has been found to correlate with ratios of memory to effector T cells upon administration to the patient [31]. CAR T-cells with naïve, central memory, or stem-like memory T cells phenotypes have greater in vivo longevity and ability and are associated with long-term remission [31]. Conversely, effector T cells have lower self-renewal capability and are more susceptible to exhaustion/senescence causing decreased effectiveness.

Persistent antigen stimulation can lead to a gradual loss of effector functions and a loss of proliferative capacity [29]. Exhausted T cells have decreased reactivation potential, minimal responsiveness and a lack of reactivation upon immune checkpoint blockade. They are observed in higher frequencies in non-responders and are associated with poor clinical outcomes [30,31,32]. Their presence may provide insight into why a CAR T therapy may demonstrate in vivo persistence and yet fail to lower tumor burden. Exhausted T cells can be identified by the expression of inhibitory receptors, such as are PD-1, CTLA-4, TIM-3, LAG-3 and TIGIT [33,34].

For solid tumor indications, the CAR T-cell clones need to traffic to the extravascular or intracerebral tumor site in order to exert their effector functions. Expression of chemokine receptors CD183 (CXCR3) and CXCR4 indicate higher migratory potential towards inflamed sites and homing ability, respectively.

### 2.2. CAR T-Cells Identification

A variety of methods are used for the specific detection of CAR T-cells by flow cytometry. If available, anti-idiotypic monoclonal antibodies (mAb) specific to the ScFv region CAR T-cells can be used. While this method is highly specific, it requires the development of an anti-idiotypic mAb, which can be very time consuming. Fortunately, there are some commercially available mAb. Anti-FMC63 mAb recognize most CD19-directed CAR T-cells, including the FDA-approved Kymriah and Yescarta, whereas anti-G4S and anti-Whitlow/218 recognize commonly used linkers for a wide variety of CAR T-cell [35,36].

Other specific methods for CAR T-cell detection include using CAR antigen labeled with a fluorescent probe, biotin or other tags, such as His. For Bi-cistronic CAR T-cell, a combination of detection approaches could be used. A study from Cordoba et al. highlights this approach in the simultaneous detection of CD19 and CD22, where CD19 is detected using the anti-idiotype HD37 labeled with PE and CD22 is conjugated to biotin and detected using a streptavidin conjugated with AF647 [37,38]. Bi-tandem CAR T-cells would be expected to have uniform expression of both CAR products; thus, they could be detected with a single method. Spiegel et al. detected their bi-tandem CD19/CD22 CAR T-cells using a fluorophore conjugated anti-idiotypic for the CD19 CAR [29].

### 2.3. Measuring Antigen Expression

The level of CAR antigen expression on the transduced T-cells should be measured, as it correlates with clinical efficacy [39]. In addition, the level of target antigen expression should be measured, as it is often one of the enrollment criteria for CAR T-cell clinical trials and has been reported to correlate with treatment response and duration. Whether in the context of clinical trials or in clinical practice, antigen expression levels of the CAR or target antigen must be quantified. Neither qualitative estimates of antigen density (e.g., 1+, 2+, etc. or dim, moderate, bright), nor arbitrary units of fluorescence intensity without any traceable calibration, should be reported. Two robust methods for antigen expression quantification are discussed in a recent paper by Tian et al. [40]. One method, first introduced in 1998, uses fluorescent quantitation beads and PE conjugated mAb at a 1:1 molar ratio to calculate antibody bound per cell (ABC) [41]. The second method uses a ratiometric comparison of the targeted cell population to the CD4 expression on autologous helper T cells using an assumed 40,000 CD4 mAb binding sites per cell [42,43]. The same paper also emphasized the requirement of quality control samples and quality assurance processes in order to generate reproducible results across laboratories. A global NIST led consortium on antigen quantitation and method calibration currently has ongoing studies into preferred methods for fluorescence calibration [13].

### 2.4. Measuring Endogenous Immune Cells

The same extensive immunophenotyping techniques used to evaluate CAR T-cells should also be conducted on normal autologous immune cells. Comparing the activation and exhaustion status, and the distribution/balance of naïve, central memory and effector memory T-cells populations in the drug product to that of endogenous (non-CAR) T-cells provides deeper insights into the behavior of the CAR T-cells. Simultaneous monitoring of the endogenous cellular counter parts can also serve as an internal biological control.

Various endogenous immunological factors, such as myeloid-derived suppressor cells (MDSC) and regulatory T cells (Treg), inhibit CAR T-cell proliferation and function. Measuring them may help elucidate mechanisms that impact CAR T-cell persistence.

### 2.5. Absolute Counts for Cellular Therapies

While there is little controversy in the field regarding the optimal process for developing and validating complex, high-parameter, spectral flow immunophenotyping methods, when it comes to cell enumeration assays, which generate an absolute count, expressed as the number of cells per unit volume, there is a surprising lack of understanding and standardization in the field [22].

Obtaining absolute counts of lymphocyte subsets by flow cytometry has been a well-established process for many years [10]. Staining is conducted in whole blood using directly conjugated mAb followed by a red blood cell (RBC) lysis step. Typically, the RBC lysis buffers also contain fixatives. Samples are then directly acquired on the flow cytometer without any washing or centrifugation steps. This procedure is often referred to as “Lyse/No Wash” staining. It is not accurate to include any wash or centrifugation steps when reporting the absolute counts, as it is not possible to assess the cell loss that would occur during these manual processes. Quantitation is achieved when the staining is conducted in the presence of quantitation beads (validated concentrations) or when samples are acquired on an instrument that is capable of accurate volumetric measurements.

The Lyse/No Wash method is only suitable for identifying major lymphocyte subsets, such as T cells (CD3+), T helper cells (CD3+, CD4+), T cytotoxic cells (CD3+, CD8+), B cells (CD19+) and NK cells (CD3−, CD56+ and/or CD16+). For these populations, the antigen expression is by and large homogeneously expressed at moderate to bright levels and resolution is adequate without a washing step. Conversely, when evaluating heterogeneously expressed antigens and antigens shared by multiple cell types, or with higher parameter assays, it becomes necessary to include a wash step.

Flow cytometric methods for quantitation of CAR T-cells need to be highly specific in order to distinguish the CAR T-cells from autologous T cells, and highly sensitive in order to precisely measure T-cells after patients have received lymphodepleting chemotherapy, which is administered pre-dosing in order to reduce the number of competing cells, including native T-cells and suppressor cells, or when the levels of circulating CAR T-cells are low. Once infused, CAR T-cells rapidly biodistribute, leading to a transient decrease in circulating cell counts. These requirements present some major challenges. The first challenge is that in order to achieve the required level of specificity, the staining method must include a washing step, yet, as mentioned above, the enumeration assays should only be conducted using the Lyse/No Wash format. Hence, the identification and enumeration of CAR T-cells requires two individual staining panels. One panel will specifically identify the CAR T-cells using a Lyse/Wash format and report the percentage and absolute numbers of the total T cells that are CAR T-cells. Peripheral blood mononuclear cells (PBMC) or whole blood can be used for this assay. The second panel will enumerate the total number of CD3 T cells and must be conducted in whole blood or bone marrow using a Lyse/No Wash assay format. Another variable to address is whether the assay is reproduceable enough to perform at local testing sites (including staining and fixation steps) vs. centralized testing.

Although assays using the Lyse/No Wash format have obtained regulatory approval for use as in vitro diagnostic tests (IVD) for the enumeration of the major lymphocyte subsets are available commercially, there are numerous reasons why these assays not suitable for the enumeration of CAR T-cell products or post-infusion patient monitoring samples. The context of use (COU) for these assays is to determine if the major lymphocytes subsets are within normal ranges. Although the assays have regulatory approval, they lack the sensitivity and specificity required for CAR T-cell quality control. In order to achieve the required sensitivity for the enumeration panel, it is necessary to stain larger volumes of blood than the 50 μL typically used in the IVD assays. It should also be noted that these assays are six-color methods, which typically use only CD45 and light scatter properties to identify lymphocytes. Several studies have demonstrated that the specificity of the lymphocyte gate can be increased with additional markers for negative selection: CD14 to exclude monocytes and CD235a to exclude unlysed and nucleated RBC. In order to achieve the required specificity and sensitivity for CAR T-cell quality control assays, the panel should include CD3, CD14, CD45 and CD235a. The gating strategy should include a time gate so that instances of fluidics irregularity can be excluded as well as double discrimination. The IVD assays do not include these features as they have not kept up with technological advances or are obstructed by regulatory requirements. Note that the initial gates in both panels (the enumeration panel and the CAR T-cell characterization) should be the same.

$$\mathrm{Cell}\;\mathrm{abs}\;\mathrm{count}=\left(\#\mathrm{events}\;\mathrm{in}\;\mathrm{cell}\;\mathrm{gate}\;÷\#\mathrm{events}\;\mathrm{in}\;\mathrm{bead}\;\mathrm{gate}\right)\times \left(\mathrm{bead}\;\mathrm{conc}÷\mathrm{staining}\;\mathrm{volume}\right)$$

Although in other situations, a two-platform method where the absolute counts for lymphocytes are obtained from a hematology analyzer for generating absolute counts is acceptable, in this context of use, it is not. When the patients/samples are lymphodepleted, a hematology analyzer may give an error message due to the low number of lymphocytes present in the sample.

Ultimately, data from the two staining panels will be used to calculate the total number of CAR T-cells.

CAR T cell abs count=% CD3 Tcells expressing CAR Ag from Lyse Wash assay×CD3 abs count from Lyse No Wash Assay 

### 2.6. B Cell Hematological Malignancy Monitoring

The current best practices for tumor-burden measurements at infusion and MRD for the target hematological malignancy should be followed. Note that for CLSI H43, Clinical Flow Cytometric Analysis of Neoplastic Hematolymphoid Cells is currently under revision and will be a reliable resource for this information when released.

Target antigen expression should be quantified as discussed above. Surface expression of the targeted antigen is required for CAR T-cell binding and killing. The sensitivity of this assay may also be improved with washed samples. The level of target antigen expression is often one of the enrollment criteria for CAR T-cell clinical trials and has been reported to correlate with treatment response and duration [39].

## 3. Immunogenicity

The measurement of humoral immunogenicity, or anti-drug antibodies (ADA), is important in clinical trials involving biologic therapeutics including CAR T-cells. ADA not only presents a patient safety concern but can impact drug efficacy. With CAR T-cell therapies the major immunogenicity risk comes from the CAR construct, which contains murine derived elements in the extracellular scFv [44,45].

For protein-based therapies, ADA are evaluated in plate-based ligand binding assays (LBA). In a typical assay, patient serum is added to wells of a microtiter plate that has been pre-coated with the therapeutic protein. Following the requisite incubations and wash steps, bound antibody is detected with an anti-human antibody and an enzymatic, fluorescent or chemiluminescent readout.

For CAR-T cells, while the recombinant CAR construct can be used as a coating antigen using the LBA format, the recombinant protein may not fully represent the physiological state of the CAR construct, for example, tertiary structures. Thus, for CAR T-cells, ADA assays are better suited to using flow cytometry [46]. Patient serum or plasma samples are incubated with CAR-expressing cell lines. Anti-CAR antibodies, ADA, are then detected with fluorophore conjugated anti-human IgG mAb. The cells are then washed and acquired on a flow cytometer, and the signal is proportional to the antibody concentration in the sample. Specificity controls for this method should include the parent (non-transfected) cell line to account for anti-cell line antibodies. In addition, pre-dose serum should be evaluated to measure any pre-existing anti-CAR target antibody. Data can be reported as the percent positive of a population or as the expression level, provided that the results are quantified and not simply presented as MdFI. Often, the results are normalized to pre-infusion levels of response.

A tiered approach is employed to evaluate sample results: Tier 1 screening, Tier 2 confirmatory and Tier 3 titration. A set of cut points are derived from naïve samples for each tier, and the samples are assessed against the cut point for positive (greater or equal to cut point) or negative (below cut point) result.

The validation of ADA conducted on a flow cytometer should follow both the regulatory guidance for ADA assays and those specific for flow cytometric methods [23,47,48].

## 4. Summary

In order to generate valuable data, the investigator must fully understand the context-of-use for the testing. Data generated during the clinical evaluation of a new drug entity (NDE) may have several different contexts of use. Results might be used for enrollment criteria, safety testing, efficacy, pharmacokinetic (PK) monitoring, or for the evaluation and/or discovery of pharmacodynamic (PD) biomarkers.

CAR T-cell clinical trials for hematological malignancies present a unique circumstance where both the NDE and the disease target are cellular populations. Hence, much of the testing for enrollment, safety, efficacy, PK and PD will be conducted on endogenous (autologous) or drug product cellular populations. Flow cytometry, the undisputed premier technology for single-cell analysis would thus be the technological platform of choice. The newest iteration of flow cytometry, spectral cytometry, offers numerous advancements that facilitate the generation of higher quality, more reliable data sets.

Additional objectives in CAR T-cell clinical evaluation are to identify correlates of response by evaluating the target cells, the endogenous immune cells as well as the CAR T-cells. These data are often used for mathematical modeling, and thus need to be highly reliable. To accomplish this, state-of-the-art methods for panel design, instrument setup and calibration and method validation must be used. Given that spectral cytometry is still relatively new, the standards and best practices are still evolving.

## Figures and Tables

**Table 1:** Comprehensive High-Parameter Panel for T cell Characterization [4,24,25,26,27,28].

Antigen	Purpose in Panel	Function
CCR10	TH subsets	Chemokine response, epithelial immunity
CD3	T cell lineage marker	T cell co-receptors, signaling
CD4	Identify CD4+ T cell subsets	Interacts with the β2-domain of MHC class II
CD8	Identify CD8+ T cell subsets	Interacts with the α3 portion of MHC class I
CD14	Monocyte lineage marker, increase purity of lymphocyte gate	Co-receptor for LPS and other microbial products
CD16	NK cells	Fc receptor FcγRIII
CD19	B cell lineage marker, increase purity of NK cell gate	B cell signaling
CD25	Treg surface staining	IL-2 receptor alpha chain
CD27	Differentiation, Co-stimulation	Co-stimulatory immune checkpoint molecule
CD28	Differentiation, Co-stimulation	T cell co-stimulatory receptor
CD38	Activation marker	Adhesion and signal transduction
CD45	Pan leucocyte marker	Signaling
CD45RA	Differentiation	Signaling
CD56	NK cells	Cell adhesion
CD62L	Differentiation	Cell adhesion, Secondary lymphoid tissue homing
CD95	Differentiation	homing
CD122	Differentiation	IL-2/IL-15 signaling
CD127	Treg surface staining, Differentiation	IL-7 signaling
CD137 (4-1BB)	Activation marker	Co-stimulatory, immune checkpoint
CD152 (CTLA-4)	Co-inhibitory	Inhibitory signaling
CD161 (KLRB1)	TH subsets, Th17 associated	Inhibitory signaling
CD183 (CXCR3)	TH subsets, Th1 associated	Leukocyte trafficking, Homing to inflamed tissues
CD185 (CXCR5)	TH subsets, Tfh associated	T cell migration to lymph nodes
CD194 (CCR4)	TH subsets, Th2 associated	Chemokine response
CD196 (CCR6)	TH subsets, Th17 associated	Chemokine response
CD197 (CCR7)	Differentiation	Chemokine response
CD223 (3 LAG-3)	Co-inhibitory	Inhibitory signaling, immune checkpoint
CD279 (PD1)	T cell exhaustion	Inhibitory signaling, immune checkpoint
CD366 (TIM-3)	Co-inhibitory	Inhibitory signaling, immune checkpoint
HLA-DR	Activation marker	MHC class II cell surface receptor, Ag presentation
KLRG1	Senescence	Inhibitory signaling, immune checkpoint
TIGIT	Co-inhibitory	Immune regulatory

**Table 2:** T cell Differentiation Phenotype [29,30].

Antigen	Naïve (Tn)	Stem-like Memory T Cells (Tscm)	Central Memory T Cells (Tcm)	Effector Memory T Cells (Tem)	Effector T Cells (Teff)
CD45RA	+++	++	++	+	−
CCR7	+++		+++	+/−	−
CD62L	+++	+++	+++	+/−	−
CD127	+/+++	+++	+++	+	+/−
CD122	+	+++	+++	+	+/−
CD28	++	+++	+++	++	−
CD27	+/−		++	++	+/−−
CD95	+/−	−	−	++	+++
KLRG1	−	−	−	+	+++

**Table 3:** T helper Subset Phenotype [25,26].

Antigen	CD4 Subset [25]					
	Th1	Th2	Th9	Th17	Th22	Tfh
CCR10					+	
CD45RA	−	−	−	−	−	−
CD161 (KLRB1)				+		
CD183 (CXCR3)	+	−	−	+/−	−	−
CD185 (CXCR5)	−	−	−	−	−	+
CD194 (CCR4)	−	+	−	+	+	−
CD196 (CCR6)	−	−	+	+	+	−

## References

[1] DePriest B.P., Vieira N., Bidgoli A., Paczesny S. (2021). An overview of multiplexed analyses of CAR T-cell therapies: Insights and potential. Expert Rev. Proteom..

[2] Xie B., Li Z., Zhou J., Wang W. (2022). Current Status and Perspectives of Dual-Targeting Chimeric Antigen Receptor T-Cell Therapy for the Treatment of Hematological Malignancies. Cancers.

[3] Hou J., Li Y., Lin Q. (2023). Bispecific antibodies and dual-targeting CAR-T cells for multiple myeloma: Latest updates from the 2023 ASCO annual meeting. Exp. Hematol. Oncol..

[4] Konecny A.J., Mage P.L., Tyznik A.J., Prlic M., Mair F. (2024). OMIP-102: 50-color phenotyping of the human immune system with in-depth assessment of T cells and dendritic cells. Cytom. Part A.

[5] Mitra-Kaushik S., Mehta-Damani A., Stewart J.J., Green C., Litwin V., Gonneau C. (2021). The Evolution of Single-Cell Analysis and Utility in Drug Development. AAPS J..

[6] Robinson J.P., Rajwa B., Gregori G., Jones J., Patsekin V. (2005). Multispectral cytometry of single bio-particles using a 32-channel detector. Advanced Biomedical and Clinical Diagnostic Systems III.

[7] Grégori G., Patsekin V., Rajwa B., Jones J., Ragheb K., Holdman C., Robinson J.P. (2011). Hyperspectral cytometry at the single-cell level using a 32-channel photodetector. Cytom. Part A.

[8] Futamura K., Sekino M., Hata A., Ikebuchi R., Nakanishi Y., Egawa G., Kabashima K., Watanabe T., Furuki M., Tomura M. (2015). Novel full-spectral flow cytometry with multiple spectrally-adjacent fluorescent proteins and fluorochromes and visualization of in vivo cellular movement. Cytom. Part A.

[9] Barnett D., Walker B., Landay A., Denny T.N. (2008). CD4 immunophenotyping in HIV infection. Nat. Rev. Microbiol..

[10] Kestens L., Mandy F. (2017). Thirty-five years of CD4 T-cell counting in HIV infection: From flow cytometry in the lab to point-of-care testing in the field. Cytometry B Clin. Cytom..

[11] Giorgi J.V., Cheng H.-L., Margolick J.B., Bauer K.D., Ferbas J., Waxdal M., Schmid I., Hultin L.E., Jackson A.L., Park L. (1990). Quality control in the flow cytometric measurement of T-lymphocyte subsets: The Multicenter AIDS Cohort Study experience. Clin. Immunol. Immunopathol..

[12] https://www.nist.gov/programs-projects/nist-flow-cytometry-standards-consortium.

[13] Gonneau C., Wang L., Mitra-Kaushik S., Trampont P.C., Litwin V. (2021). Progress towards global standardization for quantitative flow cytometry. Bioanalysis.

[14] Sommer U., Eck S., Marszalek L., Stewart J.J., Bradford J., McCloskey T.W., Green C., Vitaliti A., Oldaker T., Litwin V. (2020). High-sensitivity flow cytometric assays: Considerations for design control and analytical validation for identification of Rare events. Cytom. Part B Clin. Cytom..

[15] Looney C.M., Strauli N., Cascino M.D., Garma H., Schroeder A.V., Takahashi C., O’Gorman W., Green C., Herman A.E. (2023). Development of a novel, highly sensitive assay for quantification of minimal residual B cells in autoimmune disease and comparison to traditional methods across B-cell–depleting agents. Clin. Immunol..

[16] Kröger N., Gribben J., Chabannon C., Yakoub-Agha I., Einsele H. (2022). The EBMT/EHA CAR-T Cell Handbook.

[17] Peinelt A., Bremm M., Kreyenberg H., Cappel C., Banisharif-Dehkordi J., Erben S., Rettinger E., Jarisch A., Meisel R., Schlegel P.-G. (2022). Monitoring of Circulating CAR T Cells: Validation of a Flow Cytometric Assay, Cellular Kinetics, and Phenotype Analysis Following Tisagenlecleucel. Front. Immunol..

[18] Yakoub-Agha I., Chabannon C., Bader P., Basak G.W., Bonig H., Ciceri F., Corbacioglu S., Duarte R.F., Einsele H., Hudecek M. (2019). Management of adults and children undergoing chimeric antigen receptor T-cell therapy: Best practice recommendations of the European Society for Blood and Marrow Transplantation (EBMT) and the Joint Accreditation Committee of ISCT and EBMT (JACIE). Haematologica.

[19] Lanitis E., Dangaj D., Irving M., Coukos G. (2017). Mechanisms regulating T-cell infiltration and activity in solid tumors. Ann. Oncol..

[20] Wagner J., Wickman E., DeRenzo C., Gottschalk S. (2020). CAR T Cell Therapy for Solid Tumors: Bright Future or Dark Reality?. Mol. Ther..

[21] Owens K., Bozic I. (2021). Modeling CAR T-Cell Therapy with Patient Preconditioning. Bull. Math. Biol..

[22] Ferrer-Font L., Pellefigues C., Mayer J.U., Small S.J., Jaimes M.C., Price K.M. (2020). Panel Design and Optimization for High-Dimensional Immunophenotyping Assays Using Spectral Flow Cytometry. Curr. Protoc. Cytom..

[23] Clinical Laboratory Standards Institute (CLSI) (2021). Validation of Assays Performed by Flow Cytometry.

[24] Nettey L., Giles A.J., Chattopadhyay P.K. (2018). OMIP-050: A 28-color/30-parameter Fluorescence Flow Cytometry Panel to Enumerate and Characterize Cells Expressing a Wide Array of Immune Checkpoint Molecules. Cytom. Part A.

[25] Mousset C.M., Hobo W., Woestenenk R., Preijers F., Dolstra H., van der Waart A.B. (2019). Comprehensive Phenotyping of T Cells Using Flow Cytometry. Cytom. Part A.

[26] Stroukov W., Mastronicola D., Albany C.J., Catak Z., Lombardi G., Scottà C. (2023). OMIP-090: A 20-parameter flow cytometry panel for rapid analysis of cell diversity and homing capacity in human conventional and regulatory T cells. Cytom. Part A.

[27] Belkina A.C., Snyder-Cappione J.E. (2016). OMIP-037: 16-color panel to measure inhibitory receptor signatures from multiple human immune cell subsets. Cytom. Part A.

[28] Staser K.W., Eades W., Choi J., Karpova D., DiPersio J.F. (2018). OMIP-042: 21-color flow cytometry to comprehensively immunophenotype major lymphocyte and myeloid subsets in human peripheral blood. Cytom. Part A.

[29] Larbi A., Fulop T. (2014). From “truly naïve” to “exhausted senescent” T cells: When markers predict functionality. Cytom. Part A.

[30] López-Cantillo G., Urueña C., Camacho B.A., Ramírez-Segura C. (2022). CAR-T Cell Performance: How to Improve Their Persistence?. Front. Immunol..

[31] Fraietta J.A., Lacey S.F., Orlando E.J., Pruteanu-Malinici I., Gohil M., Lundh S., Boesteanu A.C., Wang Y., O’Connor R.S., Hwang W.-T. (2018). Determinants of response and resistance to CD19 chimeric antigen receptor (CAR) T cell therapy of chronic lymphocytic leukemia. Nat. Med..

[32] Miller B.C., Sen D.R., Al Abosy R., Bi K., Virkud Y.V., LaFleur M.W., Yates K.B., Lako A., Felt K., Naik G.S. (2019). Subsets of exhausted CD8+ T cells differentially mediate tumor control and respond to checkpoint blockade. Nat. Immunol..

[33] McLellan A.D., Rad S.M.A.H. (2019). Chimeric antigen receptor T cell persistence and memory cell formation. Immunol. Cell Biol..

[34] Gumber D., Wang L.D. (2022). Improving CAR-T immunotherapy: Overcoming the challenges of T cell exhaustion. EBioMedicine.

[35] Whitlow M., Bell B.A., Feng S.-L., Filpula D., Hardman K.D., Hubert S.L., Rollence M.L., Wood J.F., Schott M.E., Milenic D.E. (1993). An improved linker for single-chain Fv with reduced aggregation and enhanced proteolytic stability. Protein Eng. Des. Sel..

[36] Chen X., Zaro J.L., Shen W.-C. (2012). Fusion protein linkers: Property, design and functionality. Adv. Drug Deliv. Rev..

[37] Cordoba S., Onuoha S., Thomas S., Pignataro D.S., Hough R., Ghorashian S., Vora A., Bonney D., Veys P., Rao K. (2021). CAR T cells with dual targeting of CD19 and CD22 in pediatric and young adult patients with relapsed or refractory B cell acute lymphoblastic leukemia: A phase 1 trial. Nat. Med..

[38] Spiegel J.Y., Patel S., Muffly L., Hossain N.M., Oak J., Baird J.H., Frank M.J., Shiraz P., Sahaf B., Craig J. (2021). CAR T cells with dual targeting of CD19 and CD22 in adult patients with recurrent or refractory B cell malignancies: A phase 1 trial. Nat. Med..

[39] Chmielewski M., Hombach A.A., Abken H. (2010). CD28 cosignalling does not affect the activation threshold in a chimeric antigen receptor-redirected T-cell attack. Gene Ther..

[40] Tian L., Nelson A.R., Lowe T., Weaver L., Yuan C., Wang H., DeRose P., Stetler-Stevenson M., Wang L. (2024). Standardization of flow cytometric detection of antigen expression. Cytom. Part B Clin. Cytom..

[41] Davis K.A., Abrams B., Iyer S.B., Hoffman R.A., Bishop J.E. (1998). Determination of CD4 antigen density on cells: Role of antibody valency, avidity, clones, and conjugation. Cytometry.

[42] Wang L., Degheidy H., Abbasi F., Mostowski H., Marti G., Bauer S., Hoffman R.A., Gaigalas A.K. (2016). Quantitative Flow Cytometry Measurements in Antibodies Bound per Cell Based on a CD4 Reference. Curr. Protoc. Cytom..

[43] Degheidy H., Abbasi F., Mostowski H., Gaigalas A.K., Marti G., Bauer S., Wang L. (2015). Consistent, multi-instrument single tube quantification of CD20 in antibody bound per cell based on CD4 reference. Cytom. Part B Clin. Cytom..

[44] Wagner D.L., Fritsche E., Pulsipher M.A., Ahmed N., Hamieh M., Hegde M., Ruella M., Savoldo B., Shah N.N., Turtle C.J. (2021). Immunogenicity of CAR T cells in cancer therapy. Nat. Rev. Clin. Oncol..

[45] Khan A.N., Chowdhury A., Karulkar A., Jaiswal A.K., Banik A., Asija S., Purwar R. (2022). Immunogenicity of CAR-T Cell Therapeutics: Evidence, Mechanism and Mitigation. Front. Immunol..

[46] Potthoff B., McBlane F., Spindeldreher S., Sickert D. (2020). A cell-based immunogenicity assay to detect antibodies against chimeric antigen receptor expressed by tisagenlecleucel. J. Immunol. Methods.

[47] Food and Drug Administration (FDA) (2019). Guidance for Industry: Immunogenicity Testing of Therapeutic Protein Products—Developing and Validating Assays for Anti-Drug Antibody Detection.

[48] European Medicines Agency (EMA) (2017). Guideline on Immunogenicity Assessment of Therapeutic Proteins.

